# Heating of Ti_3_C_2_T_x_ MXene/polymer composites in response to Radio Frequency fields

**DOI:** 10.1038/s41598-019-52972-2

**Published:** 2019-11-11

**Authors:** Touseef Habib, Nutan Patil, Xiaofei Zhao, Evan Prehn, Muhammad Anas, Jodie L. Lutkenhaus, Miladin Radovic, Micah J. Green

**Affiliations:** 1 Artie McFerrin Department of Chemical Engineering, Texas A&M University, College Station, TX 77843 USA; 2 Department of Materials Science & Engineering, Texas A&M University, College Station, TX 77843 USA

**Keywords:** Two-dimensional materials, Chemical engineering

## Abstract

Here we report for the first time that Ti_3_C_2_T_x_/polymer composite films rapidly heat when exposed to low-power radio frequency fields. Ti_3_C_2_T_x_ MXenes possess a high dielectric loss tangent, which is correlated with this rapid heating under electromagnetic fields. Thermal imaging confirms that these structures are capable of extraordinary heating rates (as high as 303 K/s) that are frequency- and concentration-dependent. At high loading (and high conductivity), Ti_3_C_2_T_x_ MXene composites do not heat under RF fields due to reflection of electromagnetic waves, whereas composites with low conductivity do not heat due to the lack of an electrical percolating network. Composites with an intermediate loading and a conductivity between 10–1000 S m^−1^ rapidly generate heat under RF fields. This finding unlocks a new property of Ti_3_C_2_T_x_ MXenes and a new material for potential RF-based applications.

## Introduction

MXenes are a family of 2D nanosheets discovered in 2011^1,2^ with impressive functional properties that can be utilized for many applications, including catalysts, batteries, sensors, and others^2–6^. MXenes are obtained by etching out the A layer from the parent M_n+1_AX_n_ phase, where M is an early transitional metal, A is a group 13 or 14 element, X is either carbon or nitrogen, and n can be integers 1, 2, or 3. Ti_3_C_2_T_x_ is the most studied MXene (T_x_ are the terminal groups; -OH, -F, -O), derived from the etching and exfoliation of a parent Ti_3_AlC_2_ MAX phase. One of the compelling properties of Ti_3_C_2_T_x_ MXene is their high electrical conductivity, with reported values reaching 2.4 × 10^5^ S m^−1^, similar to that reported for multi-layered graphene^7^. Based on our recent reports showing that carbon nanomaterials rapidly heat in response to RF fields, we hypothesized that Ti_3_C_2_T_x_ MXenes nanosheets would respond to RF fields as well^8^.

Radio frequencies (RF) lie between 3 kHz to 300 GHz on the electromagnetic spectrum. Successful use of RF has also been demonstrated for ablation of cancerous tumors, heating food (in lieu of microwaves), curing epoxy to weld and bond materials together, and testing quality of carbon nanotube (CNT) circuits^9–20^. Our group has recently reported that nanomaterials show extraordinary heating rates in response to RF fields; Sweeney *et al*.^8^ demonstrated rapid RF heating of multiwalled carbon nanotubes embedded in epoxy resin for curing industrial grade thermoset adhesives, for which RF-curing rate was localized, volumetric, and faster than conventional oven curing^8^.

Most prior reports concerning the relationship between Ti_3_C_2_T_x_ MXenes and electromagnetic waves have focused on electromagnetic interference (EMI) shielding^21–23^. The high amount of charge carriers on the MXene surface causes the reflection of electromagnetic waves. The waves that are absorbed instead of reflected are weakened by internal attenuation between the MXene layers^21^. However, to the best of our knowledge, no one has attempted to examine the possible evolution of heat arising from Ti_3_C_2_T_x_ MXene nanosheets (in polymer composites or in a neat film) upon exposure to RF fields.

Here we demonstrate for the first time the thermal response of Ti_3_C_2_T_x_ MXene/poly(vinyl alcohol) (PVA) composites in applied RF fields in the 1–150 MHz range. PVA is a commonly used commercial polymer that is also biodegradable and hydrophilic, making it easy to process^24^. PVA was selected because of its minimal response to the RF field. In prior literature, Ti_3_C_2_T_x_/PVA composites demonstrated electrical conductivity, thermal stability, and mechanical strength^7,24,25^. We observed a high RF response for composites having conductivity in the range of 10–1000 S m^−1^ and a minimal response for composites with high and low conductivities. Using thermal imaging, we observed the heating responses of Ti_3_C_2_T_x_/polymer composites of varying compositions under low power RF waves.

## Results and Discussion

**Figure 1:**
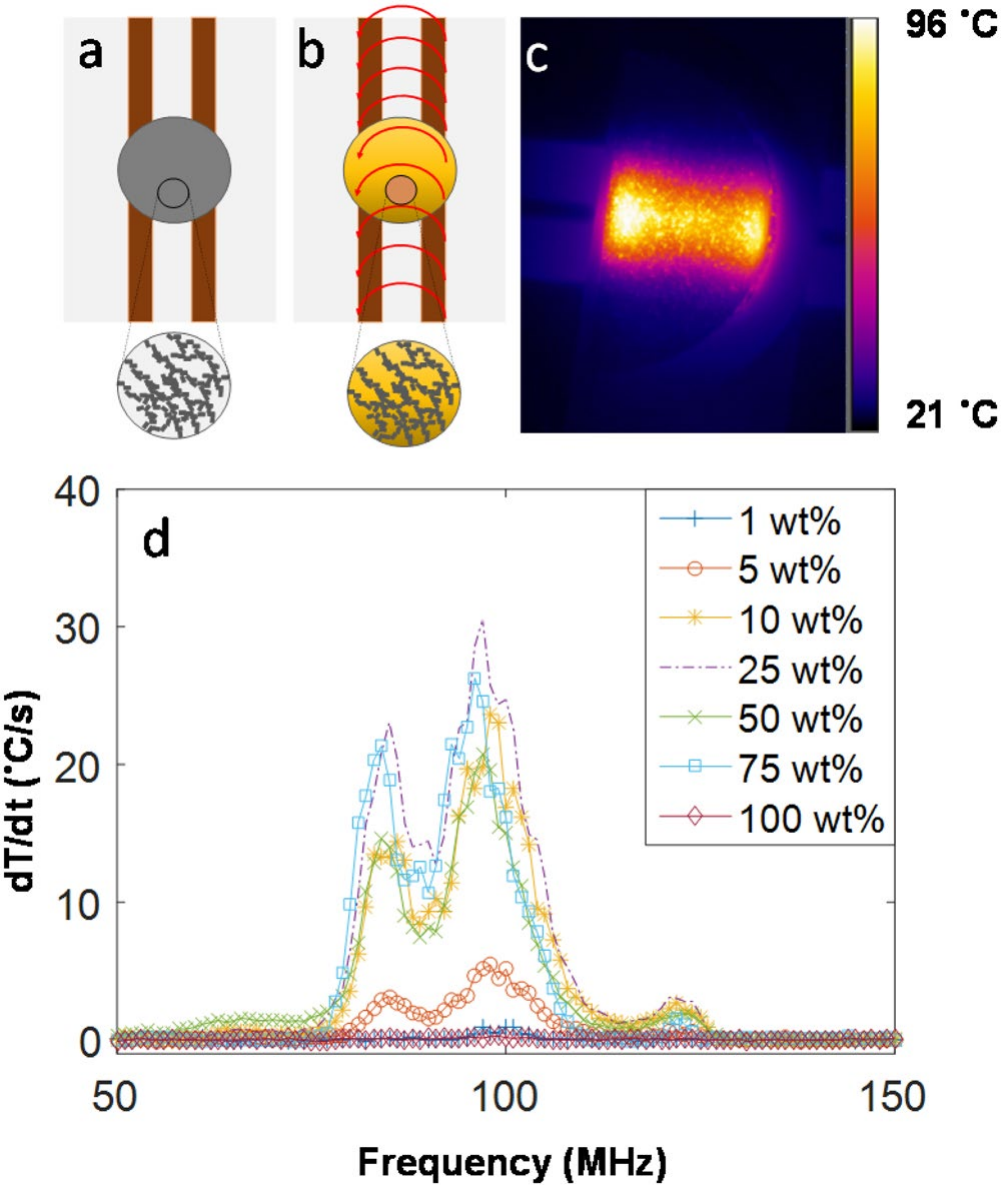
(**a**) Schematic of the RF apparatus and the Ti_3_C_2_T_x_ MXene composite sample, (**b**) same schematic but with the RF fringing field turned on which heats the sample (observed using an FLIR camera), (**c**) FLIR image of a 25 wt.% composite, and (**d**) plot of the heating rate vs. frequency to determine the resonant frequency (highest heating rate) of each sample.

Ti_3_C_2_T_x_ MXene nanosheets (Fig. S1a) were obtained using a previously reported procedure^5,26^. Ti_3_C_2_T_x_/PVA composites were prepared via vacuum filtration at initial MXene compositions of 1, 5, 10, 25, 50, and 75 wt.%; neat MXene films (100 wt.% MXene) were also prepared. Figure S1b shows a cross-sectional image of the 100 wt.% film. The sample was placed on a fringing field applicator that generated the RF field (Figs 1a,b, S2). A forward-looking infrared [thermal] camera (FLIR) was used to observe and record the heating of the MXene films.

RF heating rate varies with frequency because the impedance of the system is frequency-dependent. We utilize a frequency sweep to determine the resonant frequency has the highest resistive losses, and thus, the highest heating rate. The resonant frequency is the frequency at which the impedance of the source (RF generator) is closely matched to that of the sample, capacitor, and the connecting cables, leading to an efficient power transfer between the source and the sample^8^. The frequency sweep was programmed such that the sample was exposed to 3 W RF fields for 2 seconds (power turned on) followed by 12 seconds of cooling time (power turned off) at each frequency from 1–150 MHz (raw data shown in Fig. S3). The heating rate as a function of frequency was calculated by calculating the slope (change in temperature) within the 2 second period of power input (Fig. 1c**)**^8^.

Upon identifying the resonant frequency (98–100 MHz for all samples), the samples were exposed to RF fields at their unique resonant frequency for a 12 second period to probe their heating profiles (Figs S4 and S5). These samples were exposed to RF (at 1 W and 3 W) the same day as they were synthesized; these results are labeled as “Day 0” samples (fresh samples). A different set of samples from the same batch were stored under ambient conditions and were treated again with RF (at 1 W and 3 W) after 30 days to probe their heating profile; these results are labeled as “Day 30” samples (aged samples). The conductivity of these samples on Day 0 and Day 30 were measured using four-point probe (Table S1**)**. Additionally, neat PVA films were exposed to RF fields to determine if the matrix itself responded to RF waves, but they did not display any heating behavior (Fig. S6).

**Figure 2:**
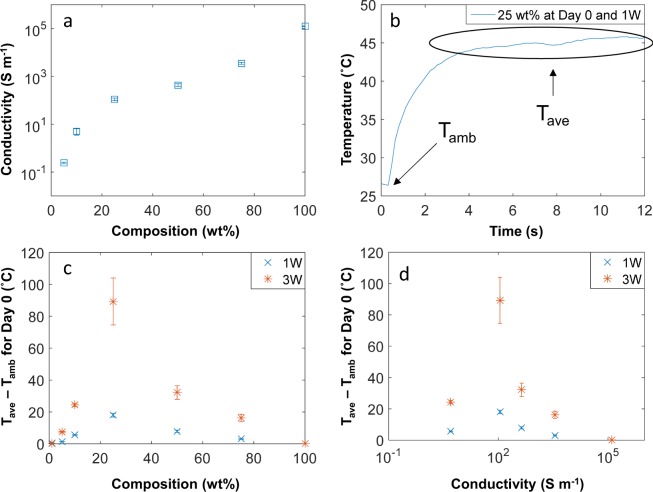
(**a**) Conductivity at each composition for Day 0 (fresh), (**b**) temperature vs time graph for 25 wt% composite sample on Day 0 and at 1 W; the error bar for each measurement is the standard deviation from T_avg_, (**c**) the rise in temperature for each Day 0 sample (at 1 W and 3 W) vs composition, (**d**) the rise in temperature for each Day 0 sample (at 1 W and 3 W) vs conductivity.

We first examine the connection between RF heating and the bulk conductivity of the samples. Conductive percolating networks in a polymer matrix are formed when the filler, in this case the nanosheets, are in close proximity to one another for the electron to transfer over (by hopping or tunneling) to the adjacent nanosheet^27^. The conductivity of the 1 wt.% composite was below the measurement threshold (<10^−3^ S m^−1^), but the 5 wt.% sample (2.41 ± 0.18 × 10^−1^ S m^−1^) was conductive as shown in Fig. 2a. This jump in conductivity from the 1 to 5 wt% composite indicates the formation of a percolating network. The 1 wt.% sample was also not responsive to RF; similar to nanocomposites with low CNT content that were also reported to be non-responsive to RF fields due to the lack of a percolating network^8,28^.

The RF-induced heating of the samples are shown by the increase in the average temperature reached during the temperature vs. time experiments (Figs S4 and S5). Figure 2b shows the temporal temperature response for the 25 wt.% composite sample at Day 0 and 1 W; the ambient temperature (T_amb_) and the average temperature (T_ave_) reached are marked. The data demonstrates that a steady temperature is eventually reached upon RF exposure after about 3–4 seconds. The temperature rise (T_avg_ − T_amb_) from RF heating of the samples as a function of composition and conductivity are shown in Fig. 2c,d, respectively. Even at different powers (1 W and 3 W), the shape of the plots in Fig. 2c,d suggests that an optimal range of conductivity (10–1,000 S m^−1^) exists for which the MXene composites will absorb RF waves and heat.

Interestingly, MXene buckypaper consisting of 100 wt.% Ti_3_C_2_T_x_ nanosheets (1.26 ± 0.03 × 10^5^ S m^−1^) did not heat under RF fields, which we attribute to reflection. As Shahzad *et al*.^21^ demonstrated, Ti_3_C_2_T_x_ surfaces possess a large number of charge carriers that are responsible for reflecting electromagnetic waves. Some of the waves that do get absorbed may experience energy loss due to attenuation within the MXene interlayer spacing^21^. Attenuation losses are also observed in the RF heating of bulk metals. This is consistent with the general observation that materials with high conductivity (>10^4^ S m^−1^) show a poor RF heating response^29,30^. Our group has observed similar trends for carbon nanotubes (CNTs) embedded in polymers, with lower microwave and RF heating response at both low and high CNT loadings. Instead, CNT/polymer composites with an intermediate loading (5 wt% CNT, 10^2^ S m^−1^) showed the highest RF heating response; our results confirm a similar trend, where an intermediate loading (25 wt% Ti_3_C_2_T_x_ MXene, 1.10 ± 0.13 × 10^2^ S m^−1^) was the most responsive^8,28^.

Another important parameter to consider is the dielectric loss tangent (tan δ); this parameter represents the ratio of electromagnetic loss over the electromagnetic storage. In other words, it is the measure of dissipation of electromagnetic energy through heat for which a material with high tan δ will dissipate more heat. Table S2 shows the tan δ of Ti_3_C_2_T_x_ MXenes and other filler materials that have been shown to heat under RF fields. Ti_3_C_2_T_x_ MXenes possess a higher dielectric loss tangent (1.5 at 2.45 GHz) than other nanomaterials, indicating that it is a good material for heat generation from the dissipation of electromagnetic waves.

**Figure 3:**
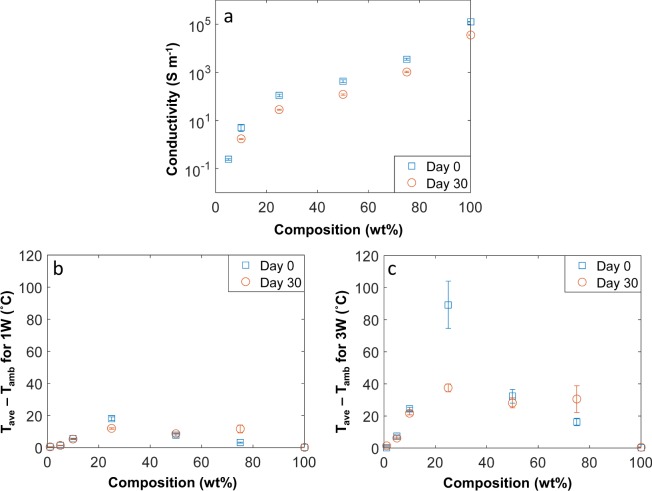
(**a**) Conductivity of Day 0 and Day 30 samples, and (**b**) temperature rise for Day 0 and Day 30 samples at 1 W, (**c**) temperature rise for Day 0 and Day 30 samples at 3 W.

We previously reported that Ti_3_C_2_T_x_ MXenes are prone to oxidation in various media; as a result, their conductivity drops over time^31^. To observe the RF response of aged Ti_3_C_2_T_x_ MXene composites, we studied samples stored in ambient conditions for 30 days. The conductivities of all samples decreased over the period of 30 days (Fig. 3a**)**, and the conductivity of the 5 wt.% composite sample by Day 30 was below the measurement threshold (<10^−3^ S m^−1^). Figure 3b,c shows that Day 30 samples possessed a similar RF heating profile to that of Day 0 samples; this again suggests that there is a range of conductivity (10–1,000 S m^−1^) where RF heating is optimal, regardless of power level. All of the Day 30 composites had a similar or lower heating value compared to the Day 0 composites. The only exception was the 75 wt.% composite. For both power values, the 75 wt.% composite experienced a jump in heating (from Day 0 to Day 30) even though the conductivity decreased. It is plausible that on Day 0, most of the RF waves reflected off the 75 wt.% sample but with a lower conductivity on Day 30, there was an increase in RF absorption leading to a higher amount of heating. There is also a significant drop in heating from the 25 wt.% sample at 3 W on Day 30, but the heating is still the highest compared to other samples; the heating trend between both power remain consistent. Our XPS results (Fig. S7) also show that the TiO_2_ content increased from 5% up to 30% for the 100 wt.% sample, which confirms that some oxidation occurred during the 30 day period.

**Figure 4:**
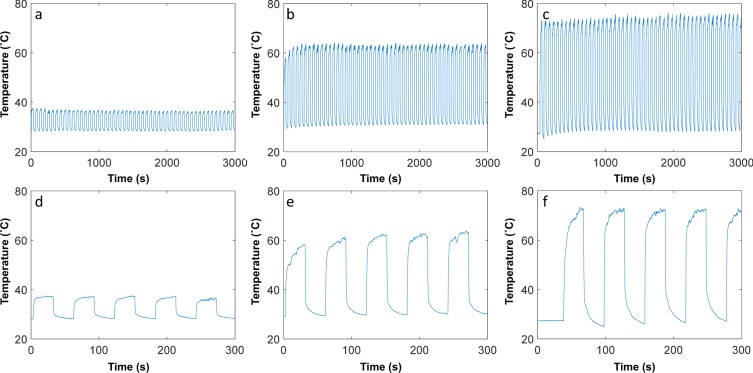
Thermal cycling protocol was 30 second with RF on followed by 30 second of RF off (1 cycle); this was repeated for 50 cycles on Day 0 at 3 W for (**a**) 5 wt.% sample, (**b**) 10 wt% sample, (**c**) 50 wt.% sample. The first 300 seconds of (**d**) 5 wt.% sample, (**e**) 10 wt.% sample, (**f**) 50 wt.% sample.

We also investigated the repeatability of RF heating over a longer time period by performing cycling experiments: One cycle consisted of the RF turned on for 30 seconds, then switched off for 30 seconds; the samples (5, 10, 50 wt.% composites) were thermally cycled for 50 cycles on Day 0 at 3 W (Fig. 4). Throughout the 50 cycles, it can be seen that the maximum temperature reached during RF heating remained relatively constant. The conductivities of the samples, pre- and post-thermal cycling are reported in Table S3. There is a decrease in conductivity for all the samples; however, the conductivity-drop after thermal cycling for the 10 and 50 wt.% composites are within the measurement error.

## Conclusion

We have demonstrated that Ti_3_C_2_T_x_ MXene composites heat under RF fields. The main parameter of interest is the composite’s conductivity, which depends on Ti_3_C_2_T_x_ content. This is a new feature that can be exploited to induce remote heating using low power RF fields. This feature may provide alternative processing routes for heating, curing, and bonding materials. The heating of Ti_3_C_2_T_x_ MXene composites suggests of the possibility of RF field heating for other types of MXenes. With the theoretical existence of over >200 stable MXene phases, there is an immense range of MXenes that may demonstrate even a stronger response to RF. Our future work will include testing different type of MXenes for RF response and also in different polymer matrices.

## Materials and Methods

### Synthesis of Ti_3_AlC_2_ MAX phase

Our previously reported procedure was followed to synthesize the Ti_3_AlC_2_ MAX phase^5,31,32^. Commercial Ti (44 μm average particle size, 99.5% purity), Al (44 μm average particle size, 99.5% purity) and TiC powders (2–3 μm average particle size, 99.5% purity), (all from Alfa Aesar, MA, USA), were used as starting raw materials to produce the parent Ti_3_AlC_2_ MAX phase. Ti, Al and TiC powders were first weighed to achieve a ratio of Ti:Al:C = 3.0:1.2:1.8 and mixed together using ball milling with zirconia beads at the speed of 300 rpm for 24 hours. The bulk high-purity Ti_3_AlC_2_ samples were then sintered at temperature of 1510 °C for 15 mins with a loading of 50 MPa using the Pulsed Electric Current System (PECS). To fabricate high-purity Ti_3_AlC_2_ powder, the PECSed sample was first drill-milled and then sieved in order to obtain powder with particle sizes below 44 µm^5^.

### Synthesis of Ti_3_C_2_T_x_ MXene clay

Ti_3_C_2_T_x_ MXene clay was synthesized by etching Al from the Ti_3_AlC_2_ phase using technique described by Ghidiu *et al*.^33^. Concentrated hydrochloric acid (HCl, ACS reagent, 37% w/w Sigma-Aldrich) was diluted with DI water to obtain 30 mL of 6 M HCl solution. This solution was transferred to a polypropylene beaker and 1.98 gm of lithium fluoride (LiF, 98 + % purity, Alfa Aesar) was added to it. This dispersion was stirred for 5 minutes using a Polytetrafluoroethylene magnetic stirbar at room temperature. The Ti_3_AlC_2_ powder was slowly added to the HCl + LiF solution to prevent overheating as the reaction is exothermic. The beaker was capped to prevent evaporation of water and a hole was made in the cap to avoid buildup of hydrogen gas. The reaction mixture was stirred at 40 °C for ~45 hours. The slurry product was centrifuged and washed with deionized (DI) water to remove all of the unreacted HF and water-soluble salts. This washing process was repeated until the pH of the filtrate increased to ~5. The reaction product is collected at the bottom of the centrifuge tubes and is extracted as Ti_3_C_2_T_x_ MXene clay^5^. This is an established procedure in the prior literature^5,6,31,32^.

### Intercalation and delamination of Ti_3_C_2_T_x_ MXene clay

A previously reported procedure was followed for intercalation and delamination^2,5,31^. The Ti_3_C_2_T_x_ MXene clay was intercalated with dimethyl sulfoxide (DMSO) and bath sonicated to obtain an aqueous dispersion of delaminated Ti_3_C_2_T_x_ MXenes following procedure described in more detail by Mashtalir *et al*.^34^. DMSO (ReagentPlus, >99.5%, Sigma-Aldrich) was added to Ti_3_C_2_T_x_ MXene to form a 60 mg/ml suspension followed by about 18 hours of stirring at room temperature. After intercalation, excess DMSO was removed by several cycles of washing with DI water and centrifugation at 5000 rpm for 4 hours. The intercalated Ti_3_C_2_T_x_ MXene clay suspension in deionized water was bath sonicated for 1 hour at room temperature followed by centrifugation at 3500 rpm for 1 hour to separate the heavier components^5^.

### Ti_3_C_2_T_x_/polymer composites

The method to prepare such composites was described by Habib *et al*.^31^ Ti_3_C_2_T_x_ powder and polyvinyl alcohol (89000–98000, 99 + % hydrolyzed, Sigma Aldrich) were bath sonicated for 15 minutes, then vacuum filtered on a polysulfone membrane (with pore size of 0.2 µm) to obtain a polymer composite film. All the composites were made with total mass of 10 mg; this ensure similar areal density for all composites. The composite films were vacuum dried overnight (room temperature) before their electrical conductivity was measured prior to RF experiments.

### RF experiments

The RF power source was a signal generator (Rigol Inc., DSG815) and 500 W amplifier (Prana R&D, GN500D. The experimental setup is shown in Fig. S2. We used a non- contact fringing-field RF applicator. It comprised of two parallel copper strips with a 2 mm spacing on a Teflon slab. All composite films were placed on a 1 mm thick glass slide to prevent any damage to RF applicator. We monitored the temperature profile using a Forward-Looking Infrared camera (FLIR systems Inc., A655sc). Heating rates as a function of frequency was determined to obtain the resonant frequency. The samples were exposed to RF ON state (power = 3 W) for 3 s followed by off state (0.0001 W) for 12 s between frequency range of 1 MHz to 150 MHz. The on and off type sweep was used to instantaneously heat the sample followed by gradual cooling at each frequency. The frequency sweep data was analyzed to generate plots of dT/dt verses frequency. We selected the resonant frequency for our heating experiments where dT/dt response was maximized. RF heating response of the films was measured at resonant frequency for 3 W and 1 W RF power. Thermal cycling experiments were performed on films that showed RF response. RF power was switched on (3 W) and off (0.0001 W) for 30 s respectively for 50 cycles at resonant frequency. These RF experimental methods were adapted from our group’s prior studies in this area^28,35,36^.

### Characterization

4 Point Resistivity Probe powered by Keithley 2000, 6221, and two 6514 were used for electrical conductivity measurements. X-ray photoelectron spectroscopy (XPS) measurements were obtained using Omnicron XPS. Malvern Zetasizer ZS90 was used to ascertain zeta potential of colloidal solutions. UV-vis measurements were obtained with Shimadzu UV-vis 2550. Scanning electron microscope (SEM) images were taken with JEOL JSM-7500 L.

## Supplementary information

Supplementary Info
