# Supplementary material for: Heating of Ti3C2Tx MXene/polymer composites in response to Radio Frequency fields
Source: Sci Rep. 2019 Nov 11;9:16489. doi: 10.1038/s41598-019-52972-2 (PMC6848125; doi:10.1038/s41598-019-52972-2)
Supplement: Supplementary file 1 — Supplementary Info [file 41598_2019_52972_MOESM1_ESM.docx]

**Title:** Heating of Ti_3_C_2_T_x_ MXene/polymer composites in response to radio frequency fields

**Authors:** Touseef Habib^a^, Nutan Patil^a^, Xiaofei Zhao^a^, Evan Prehn^b^, Muhammad Anas^a^, Jodie L. Lutkenhaus ^a,b^, Miladin Radovic^b^, and Micah J. Green^a,b*^

^a^ Artie McFerrin Department of Chemical Engineering, Texas A&M University, College Station, TX 77845

^b^ Materials Science & Engineering Department, Texas A&M University, College Station, TX 77845

*corresponding author: [micah.green@tamu.edu](mailto:micah.green@tamu.edu)

**Supplementary information**

**Figure S1**: a) SEM image of delaminated Ti_3_C_2_T_x_ MXene flake, and b) cross-sectional SEM of 100 wt.% MXene film. Films were prepared by vacuum filtering MXene dispersion.

**Figure S2**: Photo of a) the fringing field applicator and the sample; the fringing field applicator generates the RF field, and b) the FLIR camera and the RF signal generator.

**Figure S3**: Raw data generated by the RF frequency sweeps. The RF fields were turned on at 3W for 2 seconds then turned off (0W) for 13 s for each frequency from 1-150 MHz.

**Figure S4**: Raw data of temperature vs. time for a) Day 0 samples at 1W, and b) Day 0 samples at 3W.

**Figure S5**: Raw data of temperature vs time for a) Day 30 samples at 1W, and b) Day 30 samples at 3W.

**
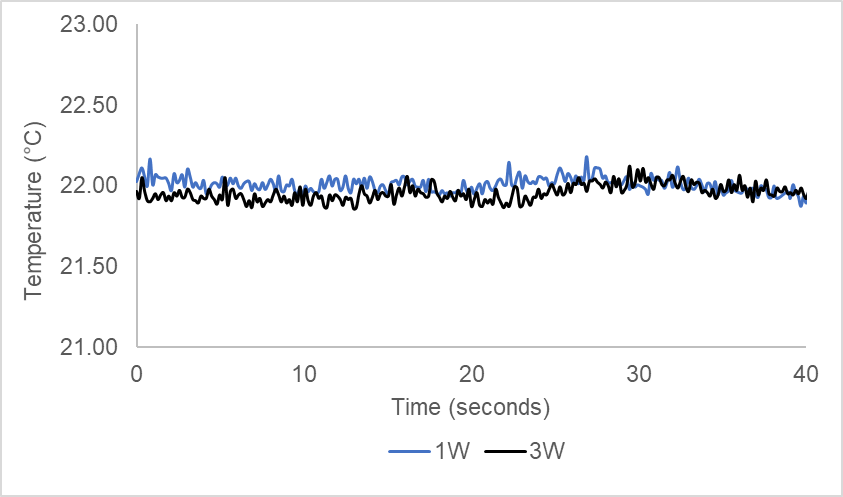
**

**Figure S6:** PVA heating behavior under RF fields (1W and 3W) at a frequency of 100 Mhz (resonant frequency)

**Table S1:** Conductivity of the samples by composition at Day 0 and Day 30.

| **MXene composition (wt.%)** | **Conductivity (S/m) Day 0** | **Conductivity (S/m) Day 30** | **% drop from Day 0 to 30** |
| --- | --- | --- | --- |
| 100 | 1.26 ± 0.03x10^5^ | 3.58 ± 0.24x10^4^ | - 71.46% |
| 75 | 3.54 ± 0.33x10^3^ | 1.05 ± 0.06x10^3^ | - 70.41% |
| 50 | 4.32 ± 0.57x10^2^ | 1.18 ± 0.07x10^2^ | - 72.56% |
| 25 | 1.10 ± 0.13x10^2^ | 2.82 ± 0.12x10^1^ | - 74.47% |
| 10 | 5.01 ± 1.51x10^0^ | 1.69 ± 0.01x10^0^ | - 66.24% |
| 5 | 2.41 ± 0.18x10^-1^ | < 10^-3^ | N/A |
| 1 | < 10^-3^ | < 10^-3^ | N/A |

**Table S2**: List of tan δ values at 2.45 GHz for different filler materials.

| **Nanomaterial** | **tan δ at 2.45 GHz** |
| --- | --- |
| Multi-walled CNTs[^1^](#_ENREF_1) | 0.25-1.14 |
| Carbon black[^1^](#_ENREF_1) | 0.35-0.83 |
| Graphene[^2^](#_ENREF_2)* | 0.06-0.09 |
| Ti_3_C_2_T_x_ MXene^[3](#_ENREF_3)^ | ~ 1.5 |

*data only available at 7.44 GHz

**Figure S7**: Ti_3_C_2_T_x_’s Titanium X-ray photoelectron spectroscopy (XPS) of a) Day 0 sample where the TiO_2_ content is roughly 4.5% and b) Day 30 sample where the TiO_2_ content is roughly 30%.

**Table S3**: Conductivities before and after thermal cycling on Day 0 at 3 W for5 wt%, 10 wt%, 50 wt% sample.

| **MXene composition** | **Pre - thermal cycling** | **Post - thermal cycling** | **% drop in conductivity** |
| --- | --- | --- | --- |
| 50 wt% | 4.32 ± 0.57x10^2^ | 3.59 ± 0.81x10^2^ | - 16.73% |
| 10 wt% | 5.01 ± 1.51x10^0^ | 4.80 ± 0.99x10^0^ | - 4.17% |
| 5 wt% | 2.41 ± 0.18x10^-1^ | 1.26 ± 0.05x10^-1^ | - 47.89% |

**References**

(1) Menéndez, J. A.; Arenillas, A.; Fidalgo, B.; Fernández, Y.; Zubizarreta, L.; Calvo, E. G.; Bermúdez, J. M. Microwave heating processes involving carbon materials. *Fuel Processing Technology* **2010**, *91*, 1-8.

(2) Rigosi, A. F.; Glavin, N. R.; Liu, C.-I.; Yang, Y.; Obrzut, J.; Hill, H. M.; Hu, J.; Lee, H.-Y.; Hight Walker, A. R.; Richter, C. A.; Elmquist, R. E.; Newell, D. B. Preservation of Surface Conductivity and Dielectric Loss Tangent in Large-Scale, Encapsulated Epitaxial Graphene Measured by Noncontact Microwave Cavity Perturbations. *Small* **2017**, *13*, 1700452.

(3) Zhao, G.; Lv, H.; Zhou, Y.; Zheng, X.; Wu, C.; Xu, C. Self-Assembled Sandwich-like MXene-Derived Nanocomposites for Enhanced Electromagnetic Wave Absorption. *ACS Applied Materials & Interfaces* **2018**, *10*, 42925-42932.
